# Fast Decision Algorithms in Low-Power Embedded Processors for Quality-of-Service Based Connectivity of Mobile Sensors in Heterogeneous Wireless Sensor Networks

**DOI:** 10.3390/s120201612

**Published:** 2012-02-07

**Authors:** María D. Jaraíz-Simón, Juan A. Gómez-Pulido, Miguel A. Vega-Rodríguez, Juan M. Sánchez-Pérez

**Affiliations:** Department of Technologies of Computers and Communications, Polytechnic School, University of Extremadura, Campus Universitario s/n, 10003 Cáceres, Spain; E-Mails: mariajaraiz@unex.es (M.D.J.-S.); mavega@unex.es (M.A.V.-R.); sanperez@unex.es (J.M.S.-P.)

**Keywords:** heterogeneous wireless sensor networks, mobility, vertical handoff, quality of service, fitness, embedded processors, FPGA

## Abstract

When a mobile wireless sensor is moving along heterogeneous wireless sensor networks, it can be under the coverage of more than one network many times. In these situations, the Vertical Handoff process can happen, where the mobile sensor decides to change its connection from a network to the best network among the available ones according to their quality of service characteristics. A fitness function is used for the handoff decision, being desirable to minimize it. This is an optimization problem which consists of the adjustment of a set of weights for the quality of service. Solving this problem efficiently is relevant to heterogeneous wireless sensor networks in many advanced applications. Numerous works can be found in the literature dealing with the vertical handoff decision, although they all suffer from the same shortfall: a non-comparable efficiency. Therefore, the aim of this work is twofold: first, to develop a fast decision algorithm that explores the entire space of possible combinations of weights, searching that one that minimizes the fitness function; and second, to design and implement a system on chip architecture based on reconfigurable hardware and embedded processors to achieve several goals necessary for competitive mobile terminals: good performance, low power consumption, low economic cost, and small area integration.

## 1. Introduction

The use of Wireless Sensor Networks (WSN) has increased substantially in the last years [1]. Both boom of this technology and its versatility have favored the appearance of applications in civil areas (industrial control, environmental monitoring, intensive agriculture, fire protection systems, and so on) and military areas (rescue operations, surveillance, *etc.*). On the other hand, the general advances in networks and communications are being constantly applied to WSN, with new ways to implement WSN services and applications appearing. Under the convergence of these technologies (WSN networks, and communications) mobility is an interesting feature that emerges with many possibilities: dynamic sensor networks, mobility of sensors, *etc.* A few cases are exposed next.

### 1.1. Mobility in Wireless Sensor Networks

There are many cases where mobility is an important feature to be taken into account in WSNs. For example, the position of mobile sensors in a WSN must be determined because the performance of event detection and tracking highly depends on the exact location information of the events that must be reported along with the event features [2,3]. Also, mobile sensors are used to assist in the initial deployment of a WSN [4], because they can move to locations that meet sensing coverage requirements. Other interesting application of the mobile sensors is the intrusion detection [5], because mobile sensors can improve barrier coverage against moving intruders. Therefore mobility applied to WSN is an interesting research topic with many fronts, being tackled along the academic and research world. For example, the NASA Glenn Research Center [6] researches about mobile platforms for sensor placement, space exploration, environmental monitoring for situation assessment, mobile networking technology applicable to mobile sensor platforms, *etc*. One important aspect to be researched is the connectivity of mobile sensors with heterogeneous wireless networks, taking into account the provided quality of service: this is the area where we place our work.

### 1.2. QoS Based Connectivity in Heterogeneous Wireless Sensor Networks

When a mobile wireless sensor (MS) can link to more than one router of the same network, we can establish a similarity to the well-known procedure of Horizontal Handoff or horizontal handover (also known as intrasystem handoff or handover) in general wireless networks. Horizontal handoff (HH) involves the change of the link of a mobile node to other wireless access points using the same technology. Nevertheless, the increasing complexity of the WSNs and their associated technologies forces us to consider heterogeneous scenarios where more than one network are present and where the nodes can be of different natures, technologies and behaviors. In these cases, and following with the previous similarity, we can follow a scheme of Vertical Handoff (VH) where the mobile node can link to other wireless access points of the same or different sensor networks and technologies.

In this work we study the more general and complex case: the VH process of a mobile wireless sensor moving along heterogeneous wireless sensor networks. In order to explain better the framework of our study, let us consider an example next.

**Figure 1.**
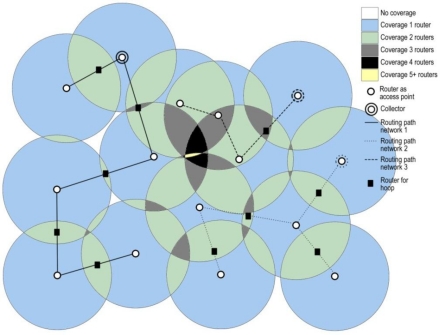
Heterogeneous wireless sensor network with three networks based on sensor routers of different technologies, giving different quality of service, and ready to provide support to mobile wireless sensors.

Figure 1 shows a case where three different networks are present. Each network has a set of routers as access points to the network for mobile wireless sensors, and a collector node which receives the information of the sensor and supplies it with different services. All the routers in this scenario could be sensors too, configuring thus a heterogeneous wireless sensor network of fixed routers (with different routing paths) ready to provide support to mobile wireless sensors where the connections are established under Quality of Service (QoS) requirements.

**Figure 2.**
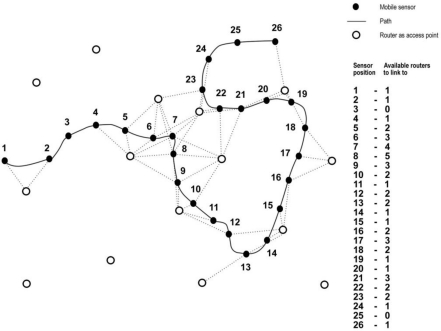
A mobile wireless sensor configures a path where several routers belonging to different heterogeneous networks can be reached in order to establish a link. This scheme is based on the scenario of Figure 1. The first column indicates the mobile sensor position in the path; the second column tells us how many available routers can be reached.

**Figure 3.**
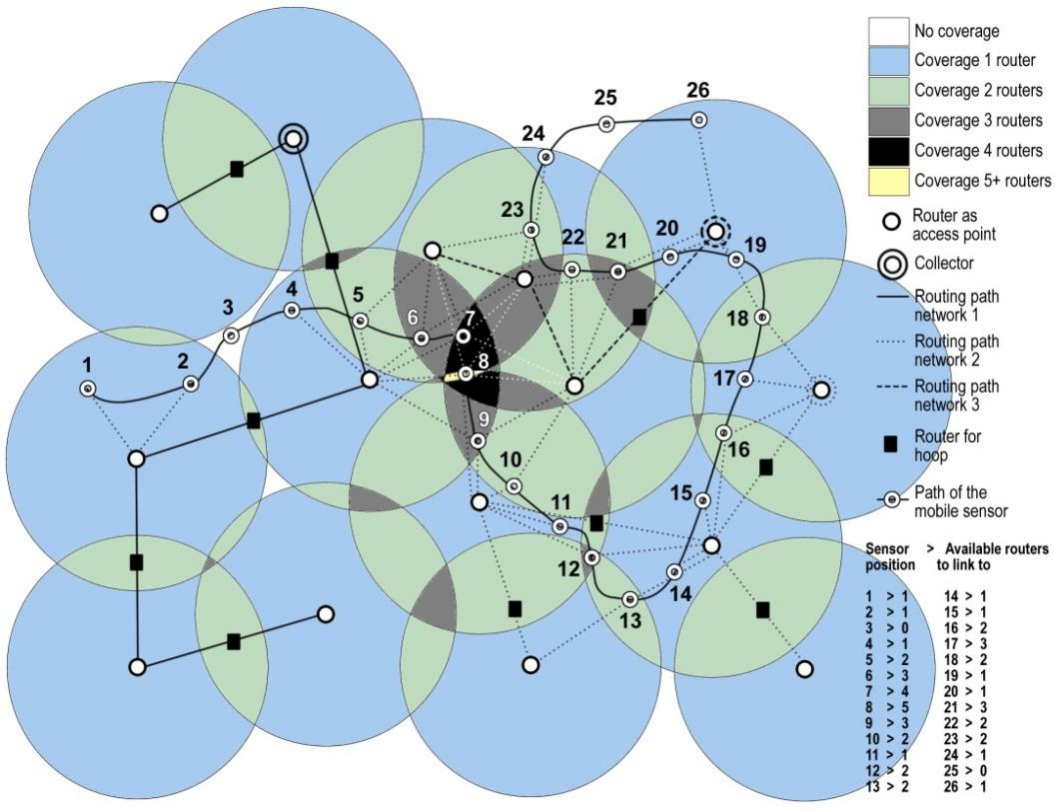
The complete scenario for the heterogeneous wireless sensor network where different processes for vertical and horizontal handoffs can be given.

Let us suppose a mobile wireless sensor moving through this scenario (Figure 2). In its route, some router nodes are within its reach, where these routers can belong to different networks. In this situation, the mobile wireless sensor must decide at any time what the best router is to connect to, in other words, what network provides it the best quality of service. As we can see in Figure 2, the mobile sensor can find more than one router in determined times, where the possible routers can be of the same network (horizontal handoff) or different networks (vertical handoff). For this case, some situations take place. For positions #3 and #25 there is not possible to link to any router, the terminal falls inside areas without coverage and the transmitted values by the mobile sensor (for example temperature) are not collected in no way. Vertical handoff happens, for example, in position #8, where three different networks can be reached because there are up to five routers belonging to these networks in the coverage area of the mobile sensor; in this case, the mobile sensor must decide what network to link to, according to the quality of service provided. On the other hand, horizontal handoff takes place, for example, in position #16, where two different routers belonging to the same network can be reached; in this case, the sensor continues connected to the current network. Figure 3 shows the complete scenario, combining Figures 1 and 2, in order to have a complete sight of the possible vertical and horizontal handoff processes.

### 1.3. Vertical Handoff

Let us explain the VH process more in detail. When the MS changes its connection from a router (access point to the network) to another one belonging to a different network, a VH can happen; if both routers are of the same network, it is a HH [7]. In any case, this is possible because the MS can have multiple interfaces to establish connections with different types of networks. In this work we have considered the VH when the QoS requirements are the factors to choose the best network to connect to. In the VH process the first phase, periodically invoked, consists of discovering which networks can be used within the coverage area and which are their services. After this, the MS determines if the connection should whether to go on the same network or switch to other one. This decision phase depends on several parameters like type of application, minimum bandwidth, access cost, transmitted power, *etc.* [8]. This is the reason to consider those parameters providing QoS to make a good choice. Finally, in the execution phase the connections are routed from the current network to the targeted network, by means of a set of tasks (authentication, data transfer, *etc.*).

In traditional handoffs only signal strength and channel availability are considered. In the new generation networks new metrics have been proposed [9]: Service type, cost, network conditions (including traffic, available bandwidth, network latency, packet loss), system performance (considering channel propagation characteristics, path loss, signal-to-noise ratio, bit error rate, battery power), mobile node conditions (like velocity, moving pattern, moving histories, location information) and user’s preferences. In this context, a VH decision metric is needed. A fitness function F can be used for handoff decision. This function evaluates the network performance based on user’s preferences [10] and evaluates various metrics [9]. Thus, the fitness function for heterogeneous networks can take into account two dimensions: The types of services requested by the user and the fitness to the network according to specific parameters, such as bandwidth, power consumption, *etc.*

Some references use the term “cost function” [9] (which must be minimized) and other ones use the term “figure of merit” [10] (which must be maximized). Both terms are comparable; they are only different in the way we want consider to optimize. In this work we bear in mind that, from now on, the term fitness is more suitable, because we want to reserve the term cost to the economic cost of the network access.

We use the fitness function described in Equation (1), where we have *n* networks, *s* services and *i* QoS parameters; *E* is an elimination factor, *w* is a weight assigned to use the QoS parameter to perform services, *p* is the cost in the QoS parameter to carry out services, and *N* is a normalization function. In any time, the sum of weights must be equal to 1 (Equation (2)). We can simplify the problem considering only one service and rejecting the elimination factor for now Equation (3), and using the logarithmic function as normalization factor [9,10]:

(1) $$F^{n}=E^{n}\sum_{s}\sum_{i}w_{s,i}\;N(p^{n}{}_{s,i})$$

(2) $$\sum_{i}w_{i}=1$$

(3) $$F(n)=\sum_{i}w_{i}\cdot \text{ln}(p_{i}^{\prime (n)})$$

If to larger *p*, larger fitness (the fitness function gets worse), then *p’* in Equation (3) is determined as in Equation (4). This is the case, for example, of the delay or economic cost. But if to larger *p*, smaller fitness (the fitness function gets better), then Equation (5) shows how *p’* must be computed (for example, bandwidth):

(4) $$p_{i}^{\prime (n)}=p_{i}^{\left(n\right)}$$

(5) $$p_{i}^{\prime (n)}=\frac{1}{p_{i}^{\left(n\right)}}$$

### 1.4. Problem Formulation

The decision phase is the frame for the formulation of the optimization problem. The choice of a network to perform VH is a very important issue when considering QoS parameters. In this choice the key is the weight tuning, because the fitness function is very sensitive to the values of the weights. In this sense, our objective is to find an optimal solution, where each solution is a sequence of weights determining the QoS. Some techniques have been developed to adjust the weights in order to find the minimum fitness, *i.e.*, Analytic Hierarchy Process [11]. Another way is to assign directly the weights by the user when a call is initialized and after that they are dynamically tuned according to the observed QoS; but this is a subjective technique and waste the user’s time. Other ways are based on automatic procedures or policy-enabled mechanisms [12]. For example, when the mobile sensor is reaching a low level of battery, then the weight of the power consumption can be increased. Other proven algorithms for the decision phase have been of the type Multiple Criteria Decision-Making (MCDM), such as Simple Additive Weighting (SAW) and Technique for Order Preference by Similarity to Ideal Solution (TOPSIS) [8,13]. Nevertheless, we think that a good way to tackle this problem is to cover all the space of possible solutions computing as many of them as be possible in regular intervals, being this characteristic the advantage against other algorithms and the basis of our work.

### 1.5. An Embedded Architecture Proposal

We propose an embedded architecture that answers to the current trends in mobile computing: low cost, low power consumption and good performance. The HiPEAC Network of Excellence on High Performance and Embedded Architecture and Compilation specifies this trend: “*People no longer only want more features and better performance, but are increasingly interested in devices with the same performance level at a lower price. [...] The limited processor performance also reduces power consumption and therefore improves mobility [...]. This trend also has an impact on software, as it now needs to be optimized to run smoothly on devices with less hardware resources [...] This trend is also leading to computers specifically designed to have extreme low power consumption*” [14].

The low cost, low power consumption and good performance (a fast obtaining of high-precision solutions) are requirements in a small electronic device (for mobile sensor purposes) which moved us to design a custom embedded microprocessor able in reconfigurable hardware to suit the algorithm.

The MS must dynamically choose the best access network for each application flow, so the vertical handoff middleware chooses the access network according to the applications requirements, the user’s preferences and the QoS parameters of the networks. Dynamic scenarios imply rapid processing, where the configurable embedded processors have demonstrated to be a good solution [15,16]. On the other hand, a low power requirement is mandatory because the increased demand for many sensor services has effects on the battery of devices. Finally, any hardware solution must consider the low cost of their components because of the competitiveness of the existing manufacturers.

## 2. Developments

In this section we expose the main developments done and results obtained in two fronts: software (a QoS-based decision algorithm for VH) and hardware (the implementation of the algorithm in an embedded microprocessor based on reconfigurable devices).

### 2.1. SEFI, a QoS-Based Decision Algorithm for VH

**Figure 4.**
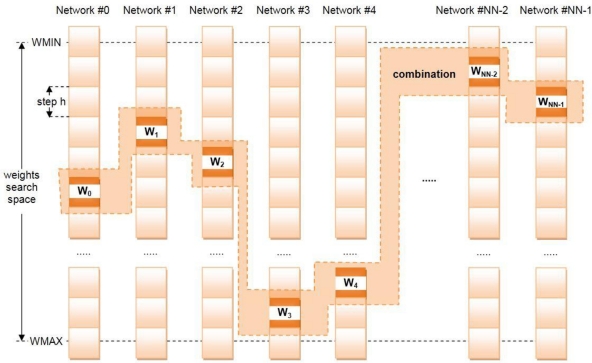
SEFI searches the best combination of QoS weights for different networks.

We name the algorithm we have developed for the VH decision phase taking into account the QoS characteristics of the networks SEFI (from “Weights Combinations Fast **SE**arch by **F**ixed **I**ntervals”). The algorithm explores the entire space of possible combinations of weights, searching those that satisfy the hard restriction imposed by (2), considering a maximum number of possible networks and a determined number of QoS parameters (NQoS) (Figure 4).

The generation of these combinations is done from a given precision value (h) and two limits determined by the user, WMIN and WMAX, where WMIN < h < WMAX, WMIN > 0 and WMAX < 1. These limits depend of the user’s profile, in other works, the purpose of the application of the mobile device in WSN. The algorithm has been programmed to perform a fast search thanks to some recursive functions. In the computation of the exhaustive search we must take into account as key parameters the precision and the number of QoS parameters considered, for a given number of networks; these parameters influence strongly the computational effort.

**Figure 5.**
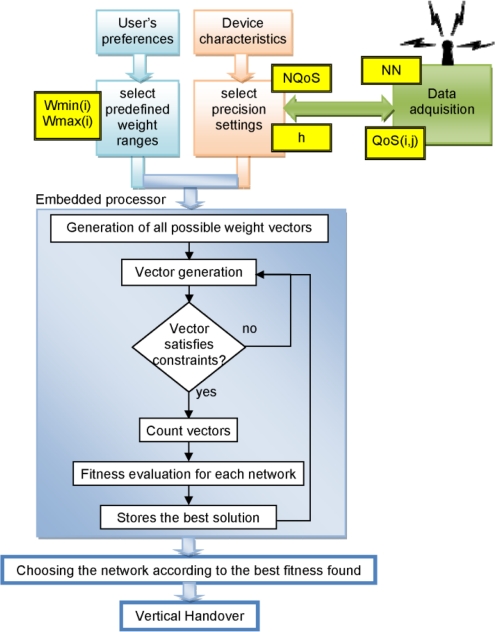
Integration and behavior of SEFI in the architecture of a MS device.

Figure 5 shows how SEFI can be integrated in a MS. The number of discovered networks and their characteristics are collected, forming the instance of the problem to be tackled by SEFI. These parameters, together with a set of predefined weight ranges (depending on the role of the MS) and the applied precision (which can be tuned depending on the size of that instance and the WSN application), make up the input to an embedded microprocessor running the SEFI algorithm. This algorithm finally selects the best solution (the network according to the best fitness found) that is used for the VH decision.

### 2.2. Instance for Experimental Purposes

We have considered a determined instance of the problem in order to validate the algorithm and perform the experiments. This instance is a scenario formed by two networks and up to four QoS parameters: throughput or bandwidth, delay, response time or latency, and cost (other QoS parameters can be easily added [17]). SEFI generates many weight vectors (solutions) {w_0_, ..., w_NQoS−1_} where NQoS is the number of QoS parameters considered in the experiments (2, 3 or 4) and the solutions satisfy the constraint given in Equation (2), exactly, without any tolerance margin. The solutions are generated by regular intervals covering all the space of solutions, with a step precision given by the variable h.

The experiments consider four possible profiles: profile #0 (all QoS parameters can have any weight from 0 to 1); profile #1 (all QoS parameters can have any weight from MINWEIGHT to MAXWEIGHT, where these variables have predefined values satisfying MINWEIGHT > 0 and MAXWEIGHT < 1); profile #2 (applications where the most important QoS parameters are delay and cost); and profile #3 (applications where the most important QoS parameter is the bandwidth). On the other hand, if NQoS = 2, we consider throughput (bandwidth) and cost; if NQoS = 3, we add delay; and if NQoS = 4, we add response time (latency). Finally, we have considered three possible precision degrees for h: 0.05, 0.01 and 0.005.

SEFI produces as output, for each profile and network, the following data: computing time, number of generated combinations, number of these combinations satisfying the restriction given in Equation (2), best fitness found and the corresponding network (that will be the solution chosen for the VH decision).

### 2.3. SEFISoC: an Embedded Microprocessor to Implement SEFI

We have used reconfigurable hardware technology in order to design and implement the SEFI digital architecture. Reconfiguration of circuitry at runtime to suit the application at hand has created a promising paradigm of computing that blurs traditional frontiers between software and hardware. This powerful computing paradigm, named reconfigurable computing [18], is based on the use of programmable logic devices, mainly field programmable gate arrays (FPGAs) [19] incorporated in board-level systems. FPGAs have the benefits of the hardware speed and the software flexibility; also, they have a price/performance ratio much more favorable than ASICs (Application-Specific Integrated Circuits). For these reasons, FPGAs are a good alternative for many real applications in image and signal processing, robotics, telecommunications, networking and computation in general. Furthermore, as the reconfigurable computing is becoming an increasingly important computing paradigm, many techniques are appearing in order to facilitate the FPGA design using embedded processors. In this line, embedded processors have been developed to bring custom processing solutions easy to program [20]. The advantages of using an embedded processor to suit the SEFI algorithm are the ones previously pointed out: good performance in an acceptable time, low power, low cost and small area, as well as the reconfigurability needed in some cases, for example firmware updates.

We have used the ISE v13.3 technology and the FPGA devices provided by Xilinx [21]. The design basically consists of an embedded microprocessor named Microblaze, with floating point unit, processor local bus and standalone operating system. Microblaze is a soft processor, that is, a processor fully customizable by the user and implementable on FPGA. The main characteristics of the implementations built in this work are listed in Table 1. Two implementations have been done: S3 (based on a Spartan3E FPGA) and V5 (based on a Virtex5 FPGA), using commercial prototyping boards (the FPGA devices used do not need neither fan nor heat skin, and external devices or peripherals are not necessary, making so possible a circuitry very reduced, necessary for mobile terminals). The first one is oriented to a very small, low power and economic device, and the second one represents a high-performance solution. The S3 implementation uses a 50 MHz clock and offers a very low on-chip power consumption of 0.01 W (estimated from the XPower analyzer tool), but it requires using an external memory because the FPGA internal resources are not able to host the SEFI code too (the external RAM chip can be easily included in the mobile sensor device). The occupied area indicates that the microprocessor can be implemented on an economic xc3s250e device (from $20). On the other hand, the V5 implementation uses a faster clock and the internal resources are enough to host both the microprocessor and the SEFI code, doing so a high-performance solution (but with an increased power consumption) interesting for some types of situations.

## 3. Experimental Results

**Figure 6.**
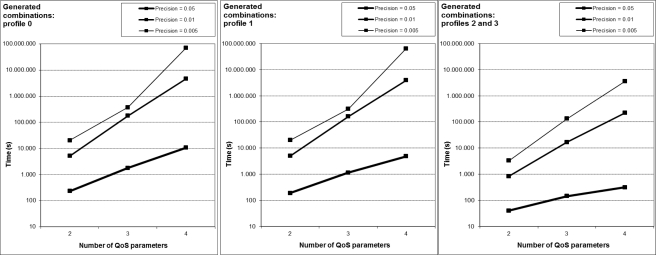
Experimental results: Generated combinations for each profile and precision.

**Figure 7.**
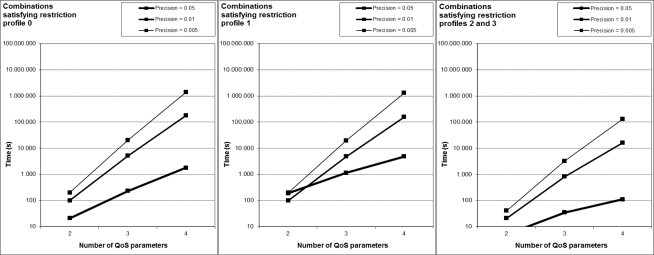
Experimental results: Combinations as solutions, for each profile and precision.

Figure 6 shows the number of generated combinations of weights for each profile and precision. For example, if we consider, h = 0.005, profile #0 and NQoS = 4, there are 70,058,751 possible combinations generated. SEFI filters the solutions that satisfy the restriction imposed by Equation (2), so there are less possible solutions to the problem than combinations generated, as we can see en Figure 7.

Following with the last example, only 1,373,701 combinations are solutions. SEFI applies the fitness function to all these solutions in order to obtain the combination with the lowest fitness value; hence its corresponding network will be used for the VH decision.

We have also observed two interesting results, after examining detailed data from many other experiments. On the one hand, the best network could be any of the considered ones in any time, showing the importance of searching within a wide space of solutions. On the other hand, when the number of QoS considered parameters grows, other network different to the best one previously found can emerge now as the best.

**Figure 8.**
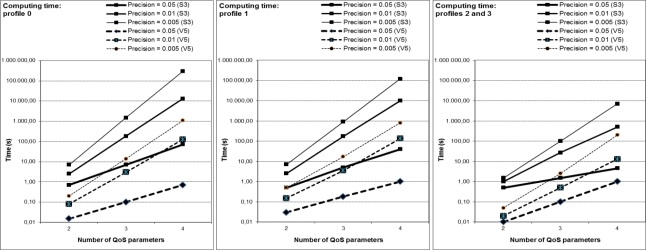
Experimental results: Computing times, for each profile, precision and FPGA.

Obtaining the best solution becomes slower to compute when considering more QoS parameters or more precision, because of the increased number of generated combinations. Figure 8 shows the computing times for both FPGA devices in all the cases, with values from milliseconds to several minutes.

Depending on the number of the discovered networks and their characteristics, the type and technology of the mobile sensor, the requirements of the application, and so on, SEFI can initially adjust the precision, profile and number of QoS parameters in order to obtain solutions in real time or in an acceptable time.

## 3. Conclusions

The aim of this paper was twofold: (1) to develop a fast algorithm to search all the possible combinations of quality-of-service weights in order to determine the best network for the vertical handoff decision performed by a mobile wireless sensor, given a determined heterogeneous scenario of wireless networks and the user’s preferences; and (2), to design and test an embedded processor with reconfigurable hardware technology to run the algorithm taking into account the constraints of the problem and the requirements needed in mobile sensor devices for dynamic environments: fast computation, small area, low power, and low economic cost. This was the first time that an algorithm for the vertical handoff decision phase based on QoS has been implemented in reconfigurable embedded processors. The results showed that the proposed architecture was able to achieve an acceptable performance.

Nevertheless, we have verified that the computing time increases a lot when we consider more networks, more quality-of-service parameters and smaller intervals searching the solutions, because the number of generated combinations becomes huge. This makes it necessary to enable mechanisms to select precisions that permit finding solutions in an acceptable time. We think this is a good starting point to add intelligent techniques to the algorithm in order to obtain good solutions in hard-computing scenarios without loss of precision or quality. This approach is our current research line.

## Figures and Tables

**Table 1.** Main characteristics of the implemented architecture.

	**S3**	**V5**
Prototyping board	Digilent Nexys2-500	Xilinx XUPV505-LX110T
FPGA	Spartan 3E: xc3s500e-fg320-4	Virtex5: xc5vlx110t-ff1136-1
Processor/memory	Microblaze with FPU and PLB/external	Microblaze with FPU and PLB/256KB local
Operating system	Standalone	Standalone
Clock frequency	50 MHz	125 MHz
Occupied slices	43%	10%
Power consumption	0.097 W	1.34 W
